# Anthrahydroquinone-2-6-disulfonate is a novel, powerful antidote for paraquat poisoning

**DOI:** 10.1038/s41598-021-99591-4

**Published:** 2021-10-11

**Authors:** Jin Qian, Chun-Yuan Wu, Dong-Ming Wu, Li-Hua Li, Qi Li, Tang Deng, Qi-Feng Huang, Shuang-Qin Xu, Hang-Fei Wang, Xin-Xin Wu, Zi-Yi Cheng, Chuan-Zhu Lv, Xiao-Ran Liu

**Affiliations:** 1 Key Laboratory of Emergency and Trauma of Ministry of Education, The First Affiliated Hospital of Hainan Medical University, Hainan Medical University, Haikou, 571199 China; 2 Institute of Environment and Plant Protection, Chinese Academy of Tropical Agricultural Sciences, Haikou, 571101 China; 3 Emergency Medicine Center, Sichuan Provincial People’s Hospital, University of Electronic Science and Technology of China, Chengdu, 610072 China

**Keywords:** Drug development, Drug therapy, Experimental models of disease

## Abstract

Paraquat (PQ) is a widely used fast-acting pyridine herbicide. Accidental ingestion or self-administration via various routes can cause severe organ damage. Currently, no effective antidote is available commercially, and the mortality rate of poisoned patients is exceptionally high. Here, the efficacy of anthrahydroquinone-2-6-disulfonate (AH_2_QDS) was observed in treating PQ poisoning by constructing in vivo and ex vivo models. We then explored the detoxification mechanism of AH_2_QDS. We demonstrated that, in a rat model, the PQ concentration in the PQ + AH_2_QDS group significantly decreased compared to the PQ only group. Additionally, AH_2_QDS protected the mitochondria of rats and A549 cells and decreased oxidative stress damage, thus improving animal survival and cell viability. Finally, the differentially expressed genes were analysed in the PQ + AH_2_QDS group and the PQ group by NextGen sequencing, and we verified that Nrf2’s expression in the PQ + AH_2_QDS group was significantly higher than that in the PQ group. Our work identified that AH_2_QDS can detoxify PQ by reducing PQ uptake and protecting mitochondria while enhancing the body's antioxidant activity.

## Introduction

Paraquat (1,1'-dimethyl-4,4'-bipyridinium, PQ) is a fast-acting herbicide widely used for chemical weed control worldwide^1^. PQ is exceptionally toxic to the human body and can cause acute poisoning by accidental or spontaneous ingestion. The vast majority of these poisonings are oral ingestions, and the adult lethal dose is 5–15 mL (20–40 mg/kg) of a 20% (w/v) aqueous solution. When PQ enters the body by various means (such as oral, local contact and injection), it is rapidly absorbed and enriched, causing an acute poisoning reaction that damages the digestive tract, kidneys, liver, lungs and other organs, resulting in multi-organ failure, with a mortality rate of 50–80%^2–5^.

Clinically, activated carbon and montmorillonite powder are commonly used via gastric administration, and 20% mannitol is used as a cathartic (“white and black” scheme)^6–8^. The above method is mainly based on the physical adsorption of PQ to accelerate excretion and prevent its further absorption. In addition, many chemical methods for the treatment of PQ poisoning have also been developed, such as vitamin C and glutathione, which are also used to combat peroxidation damage caused by PQ^9,10^. Current anti-PQ therapies include oxygen therapy, immunosuppressants, chemotherapy drugs, antifibrotic drugs, and even lung transplant surgery to manage of PQ poisoning^11–14^. Unfortunately, the clinical benefits of these technologies are insufficient, and the mortality of affected patients remains high. It is generally believed that PQ causes abundant reactive oxygen species (ROS) after absorption into the blood^15^. Once an imbalance of the redox system begins to occur, it will destroy mitochondria, causing the activity decline of various antioxidant enzymes, which are continuously stimulated by oxidation in biological systems^16^. In summary, we believe that blocking the absorption of PQ and antioxidant capacity may be key to the treatment for paraquat poisoning. Therefore, there is an urgent need for a new antidote that meets all these requirements.

Recently, we developed an antidote that can directly bind to PQ. This antidote, called anthrahydroquinone-2-6-disulfonate (AH_2_QDS)^17,18^, has strong redox properties and can quickly reduce PQ to nontoxic substances in vitro. Since ionic PQ contains a dibasic pyridinium ion structure and AH_2_QDS contains a dibasic sulfonic acid structure, both planar structures provide low steric hindrance and strong molecular attraction interactions lead to the formation of a chain-like structure, with a needle-like structure under the scanning electron microscope (Figure S5B). Early in vitro experiments found that after mixing PQ with AH_2_QDS, a green precipitate was formed immediately. If detoxification is performed at a 1:1 mol ratio, the concentration of PQ in the mixed solution will drop below the limit of detection in 60 min. Accordingly, PQ can be transformed into a nontoxic substance in the system (Figure S5A).

We hypothesised that AH_2_QDS would be able to achieve detoxification of PQ in the organism. To test this hypothesis, we constructed an in vitro toxicity model of A549 cells^19^and then intervened with AH_2_QDS, confirming that compared with the PQ group, the AH_2_QDS intervention group showed lessened functional damage mitochondria and significantly improved cell activity. After that, we established a gavage PQ poisoning SD rat model^20^ and found a significant decrease in PQ concentration in plasma detoxified with AH_2_QDS. Furthermore, compared with the PQ-treated rats, the tissue suffered only minor damage in the AH_2_QDS intervention group. The 30-day survival rate was also improved. We found AH_2_QDS restored the level of antioxidants and diminished PQ-induced oxidative stress by lowering the level of oxidative stress factors. To further explore the detoxification mechanism of AH_2_QDS, we analysed the differentially expressed genes by NextGen sequencing, and we found that oxidative stress plays an essential role in AH_2_QDS treatment of PQ poisoning and that the nuclear factor Nrf2 plays a vital role in this process.

In conclusion, AH_2_QDS can rapidly neutralize PQ to prevent the absorption of poison and remove the oxidative stress products produced by PQ, thus suggesting great clinical promise as a specific antidote for PQ poisoning.

## Results

### Binding of PQ and AH_2_QDS

**Figure 1:**
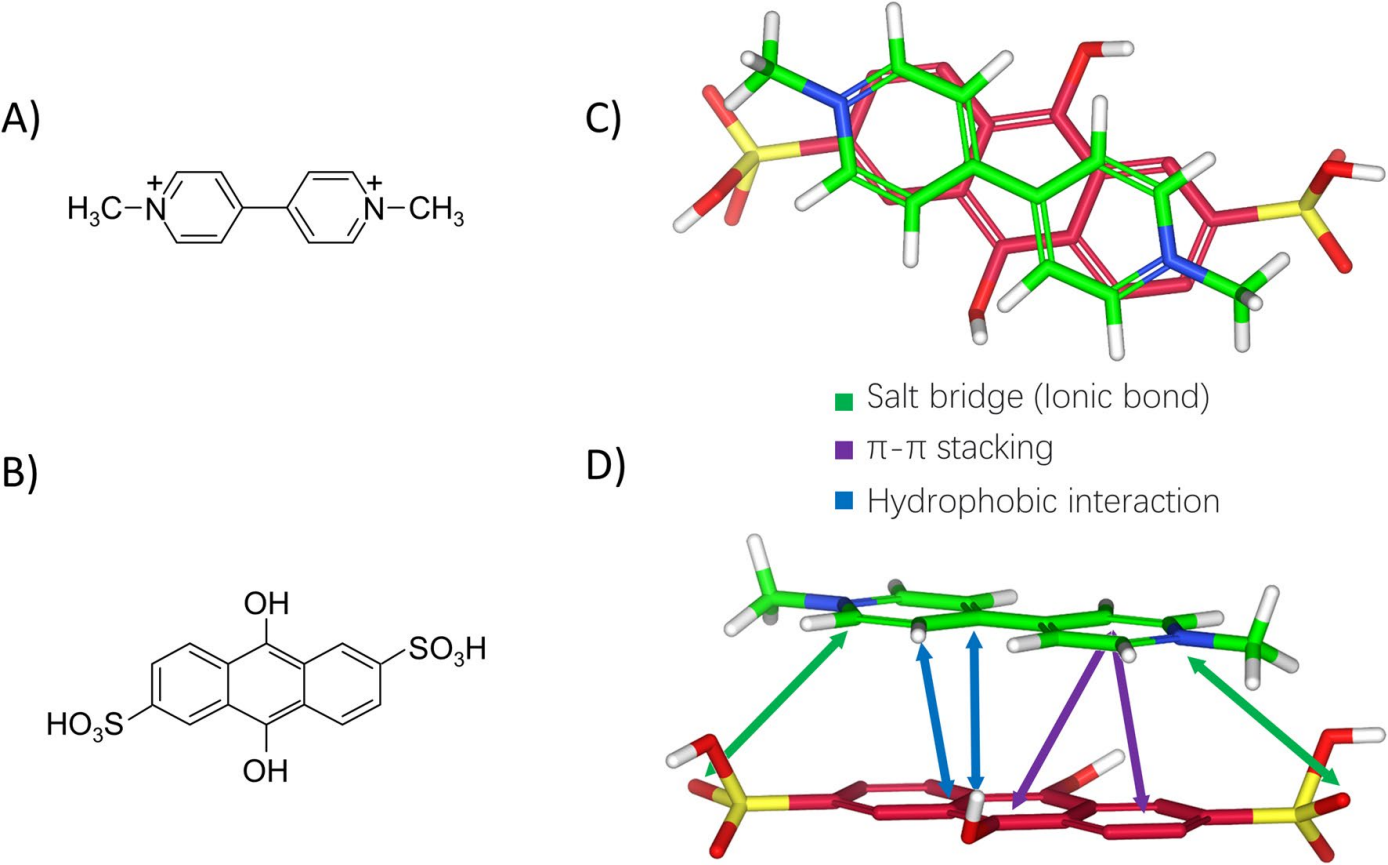
Complexation of PQ by AH_2_QDS. (**A**,**B**) Structures of PQ (**A**) and AH_2_QDS (**B**). (**C**,**D**) Complexes of PQ and AH_2_QDS by AutoDock Vina. (**C**) is the side view and (**D**) is the top view.

Firstly, we used AutoDock Vina^21^ to simulate the binding conformation between AH_2_QDS and PQ (Fig. 1A,B). A grid map of dimensions 26 Å × 26 Å × 26 Å with a grid space of 0.375 Å was set. The search space's center was set to − 0.014 Å, − 0.008 Å and − 0.037 Å (x, y, z). One hundred GA (genetic algorithm) runs was placed, and all other parameters were the default option values by AutoDock Vina. Molecular docking results indicate that the crystal structure of the PQ + AH_2_QDS complex contains three intermolecular interactions, with π π stacking between the two benzene rings of AH_2_QDS and the PQ molecule, hydrophobic interactions between the middle of AH_2_QDS and PQ, and the two sides of AH_2_QDS forming a salt bridge with PQ (Fig. 1C,D). The above docking simulation studies demonstrate at a theoretical level that AH_2_QDS is able to bind to PQ to form a complex, thereby eliminating the toxicity of PQ. Next, we constructed in vivo and in vitro models to validate the detoxification of PQ by AH_2_QDS.

### AH_2_QDS for the treatment of PQ poisoning in vitro

In the in vitro experiment, we first used CCK8 to determine the effects of different concentrations of PQ on the viability of A549 cells^22^. As shown in Figure S1A, the cell viability decreased gradually in a time-dependent manner starting 24 h after the PQ intervention. Interestingly, a significant difference in cell viability was caused by different concentrations of PQ at 48 h. At 72 h, under the 200 μM PQ intervention, the cell viability decreased to 50% (Figure S1A). According to the data, we used this condition in subsequent experiments. At the same time, we measured the effects of different concentrations of AH_2_QDS on the viability of A549 cells (Figure S1B). It is worth noting that when the concentration of AH_2_QDS is greater than 200 μM, it will also have a toxic effect on cells, so we chose 200 μM AH_2_QDS as the concentration for the follow-up experiment. Given the oxidative damage-related mechanism of PQ, we also used glutathione, which is often used to resist the damage caused by oxidative stress^10^. Here, we chose different concentrations of glutathione to determine its effect on the viability of A549 cells (Figure S1C). The results showed that glutathione had no toxic effect on cells.

**Figure 2:**
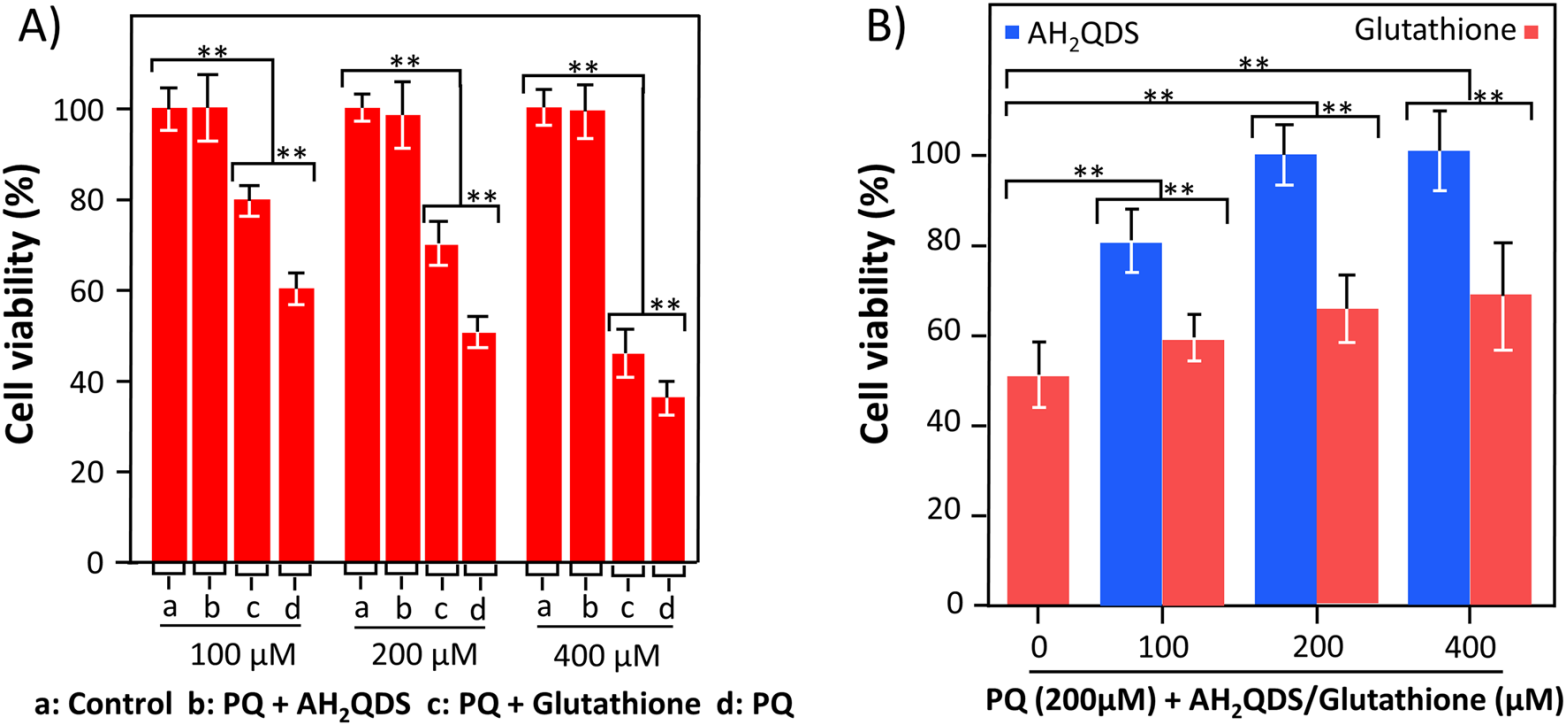
Antidotal effects of AH_2_QDS on PQ poisoning in vitro. (**A**) A549 cells in different treatment groups were incubated at different drug concentrations. (**B**) Viability of A549 cells co-cultured with PQ (200 μM) and various concentrations of AH_2_QDS/Glutathione for 72 h. Data are presented as means ± SEM, n = 3, NS = not significant, *P < 0.05, **P < 0.001.

Next, Fig. 2A,B showed that PQ could significantly damage the viability of A549 cells, while glutathione and AH_2_QDS intervention raised the activity of A549 cells. In addition, we pre-intervened AH_2_QDS and glutathione and then administered PQ staining after different treatment times to assay the cell activity for 72 h. The results showed that the Glutathione/AH_2_QDS pretreatment + PQ group still showed a significant rise in cell viability compared to the PQ group (Figure S2). However, the Glutathione pretreatment group only showed good results at 1 h, while the AH_2_QDS pretreatment group could still exert excellent cytoprotective effects until 12 h. This result reflects the ability of both AH2QDS and Glutathione to induce intrinsic cellular protective effects, however, AH_2_QDS is more protective than Glutathione.

### AH_2_QDS improve antioxidation in the treatment of PQ poisoning

**Figure 3:**
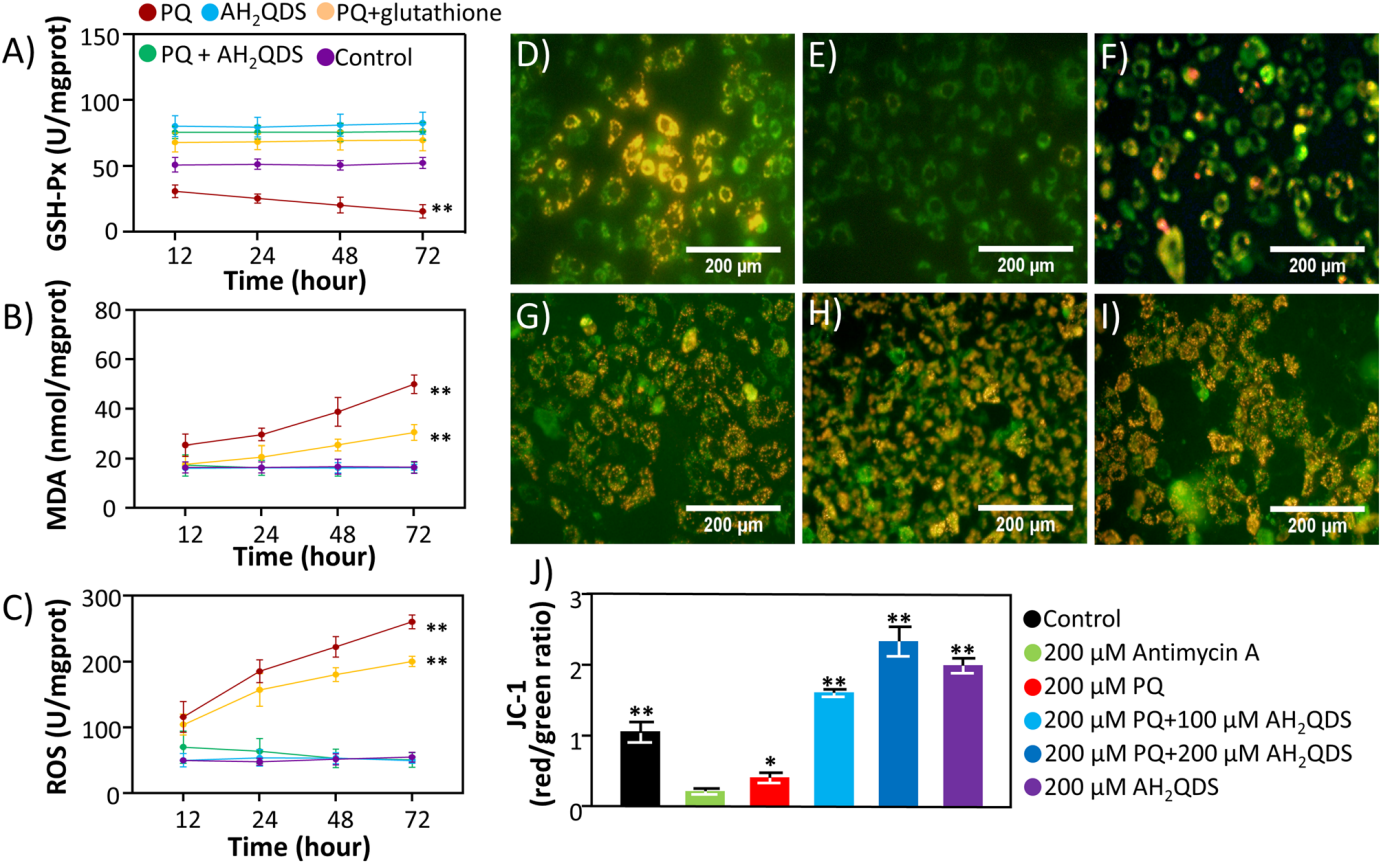
AH_2_QDS can protect the function of mitochondria and improve antioxidation in the treatment of PQ poisoning in vitro. (**A**–**C**) The levels of GSH-Px, MDA, and ROS were detected in different treatment groups. (**D**–**I**) The mitochondrial membrane potential of A549 cells in different treatment groups was detected. When the mitochondrial membrane potential is high, it can produce red fluorescence, whereas, when the level of mitochondrial membrane potential is low, it can show green fluorescence. (**D**) The control group without any handling. (**E**) For the 200 μM Antimycin A group, A549 cells were incubated with 200 μMAntimycin A. (**F**) is the 200 μM PQ group, A549 cells were incubated with 200 μM PQ. (**G**) shows 200 μM PQ + 100 μM AH_2_QDS group, A549 cells were incubated with 200 μM PQ and 100 μM AH_2_QDS. (**H**) represents 200 μM PQ + 200 μM AH_2_QDS group, A549 cells were incubated with 200 μM PQ and 200 μM AH_2_QDS. (**I**) means that in the 200 μM AH_2_QDS group, A549 cells were incubated with only 200 μM AH_2_QDS. (**J**) Mitochondrial membrane potential was determined using Mitochondrial Membrane Potential Assay Kit with JC-1. Mitochondrial JC-1 monomers (green) and aggregates (red) were observed under a fluorescence microscope. The mitochondrial membrane potential was presented as the ratio of J-aggregates to monomers. Data are presented as means ± SEM, n = 3, *P < 0.05, **P < 0.001.

PQ poisoning often causes oxidative stress damage. Consistent with the literature, the results in Fig. 3A–C showed that the level of GSH-Px in the PQ group decreased, indicating that its antioxidant capacity decreased^23^. In contrast, ROS and MDA levels increased in the PQ group, indicating that oxidative damage was aggravated^24,25^. In this context, the level of GSH-Px in the group treated with AH_2_QDS was significantly higher, and the levels of ROS and MDA were significantly lower than those in the PQ group. The same trend was also confirmed in vivo (Fig. 7D–F). In summary, AH_2_QDS plays an antioxidant role in the treatment of severe PQ.

### Protective effect of AH_2_QDS on cell mitochondria

Many studies have reported that PQ poisoning often causes damage to mitochondria^26–28^. To explore whether AH_2_QDS can protect mitochondria, we used a transmission electron microscope to observe the mitochondrial structure under a microscope. From the Fig. 8D–F, we can see that after PQ intervention, the mitochondrial structure of A549 cells was destroyed, vacuoles appeared in the cell body, the cell wall was broken, and a large number of organelles were extruded. However, the morphology of cells in the untreated control group and PQ + AH_2_QDS group was normal, the chromatin was evenly distributed, the morphology of the cells was as expected, and the morphology of the mitochondria was normal. Furthermore, through detection of the mitochondrial membrane potential, we found that the membrane potential of the PQ + AH_2_QDS high-dose group was the highest, followed by the high-dose group and PQ + AH_2_QDS low-dose group, and the mitochondrial membrane potential of the PQ group was the lowest (Fig. 3D–J). The above results indicate the protective effect of AH_2_QDS on cell mitochondria in vitro.

### The survival rate in a rat model of PQ poisoning

**Figure 4:**
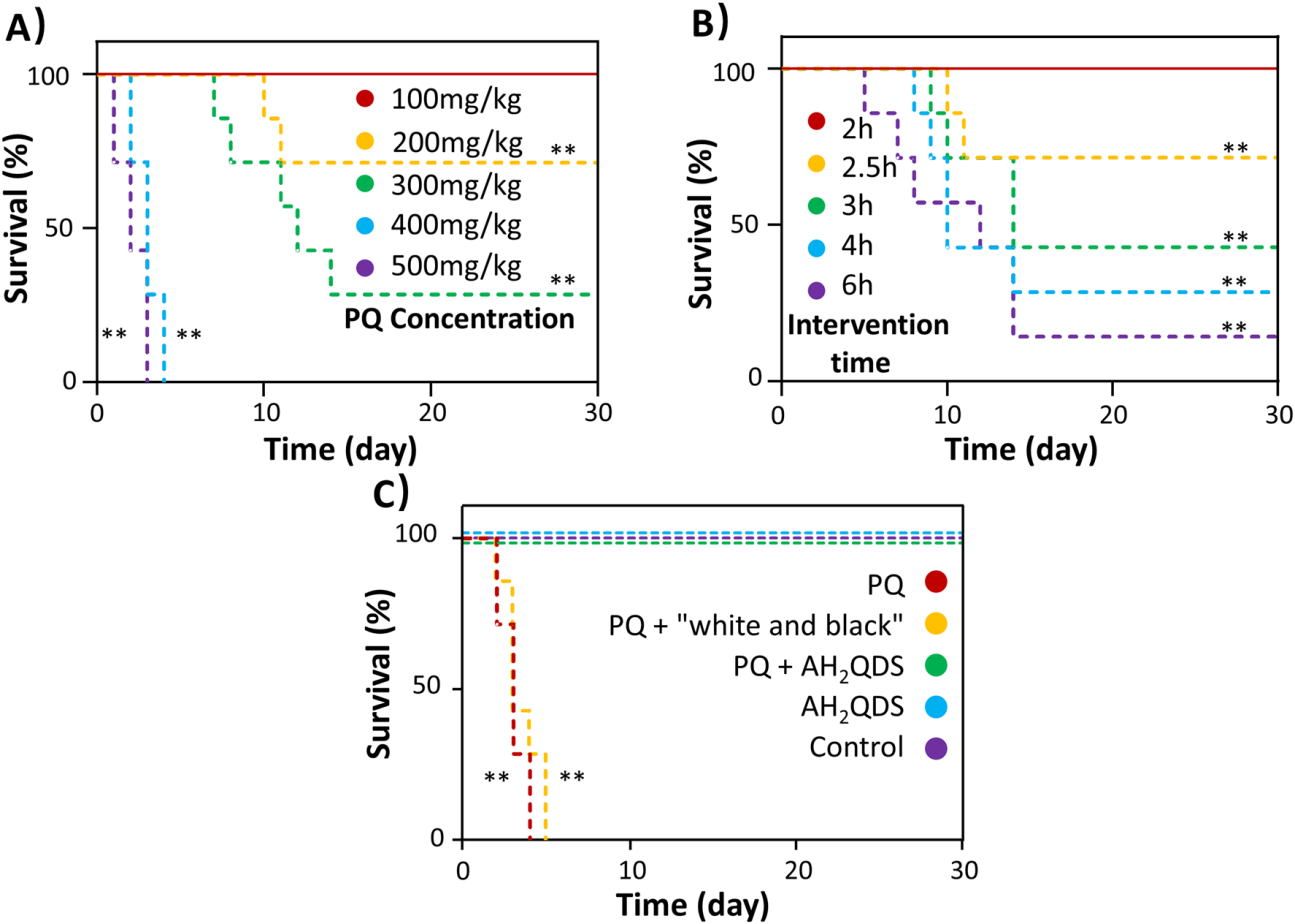
AH_2_QDS can improve the survival rate of rats with PQ poisoning. (**A**) The survival of rats with different concentrations of PQ. (**B**) Prolong the time of AH_2_QDS intervention in rats with PQ poisoning and observe the changes in survival rate. (**C**) The survival curve of rats in different treatment groups. The untreated control group did not make any interventions. In the AH_2_QDS group, only 400 mg/kg AH_2_QDS antidote was given by gavage. PQ was given by gavage only at a concentration of 400 mg/kg in the PQ group. In the PQ + "white and black" group, 400 mg/kg of PQ was given by gavage first, and 500 mg/kg was given by gavage 2 h later, with a "white and black" scheme. In the PQ + AH_2_QDS group, 400 mg/kg of PQ was given to the stomach first, and 400 mg/kg of AH_2_QDS antidote was given 2 h later. Kaplan–Meier survival analysis was used to analyze the survival rate of rats in different treatment groups, n = 7, *P < 0.05, **P < 0.001.

According to the literature, the single-dose oral LD50 for PQ was 100 mg/kg in rats^29^. Therefore, to determine PQ toxicity, we gavaged PQ at doses of 100, 200, 300, 400, and 500 mg/kg in vivo (Fig. 4A). We found that when the concentration of PQ was more than 300 mg/kg (3X LD50), the rats showed obvious poisoning symptoms and died within two weeks. When the concentration of PQ was more than 400 mg/kg (4X LD50), the animals died in approximately three days. According to these data, to show the superior detoxification ability of AH_2_QDS, we chose 400 mg/kg (4X LD50) as the dose of PQ to evaluate the detoxification effect of AH_2_QDS. Subsequently, we used AH_2_QDS to detoxify the animals at different times after exposure. As shown in Fig. 3A, the 30-day survival rate of rats exposed to 400 mg/kg PQ could reach 100% when they were detoxified with AH_2_QDS within 2 h. However, as the time window for AH_2_QDS treatment was extended, the 30-day survival rate of SD rats gradually decreased (Fig. 4B). According to the above results, we chose 2 h as the detoxification time of AH_2_QDS. Next, we designed different experimental groups to verify the detoxification effect of AH_2_QDS (Fig. 4C). We discovered that the untreated control group's 30-day survival rates, the AH_2_QDS group, and the PQ + AH_2_QDS group were all 100%. The untreated control group's 30-day survival rates and the PQ + "white and black" group were zero, and all of the rats died within one week. The detoxification effect of AH_2_QDS is better than that of the “white and black” scheme.

### AH_2_QDS mitigates organ damage in a rat model of PQ poisoning

**Figure 5:**
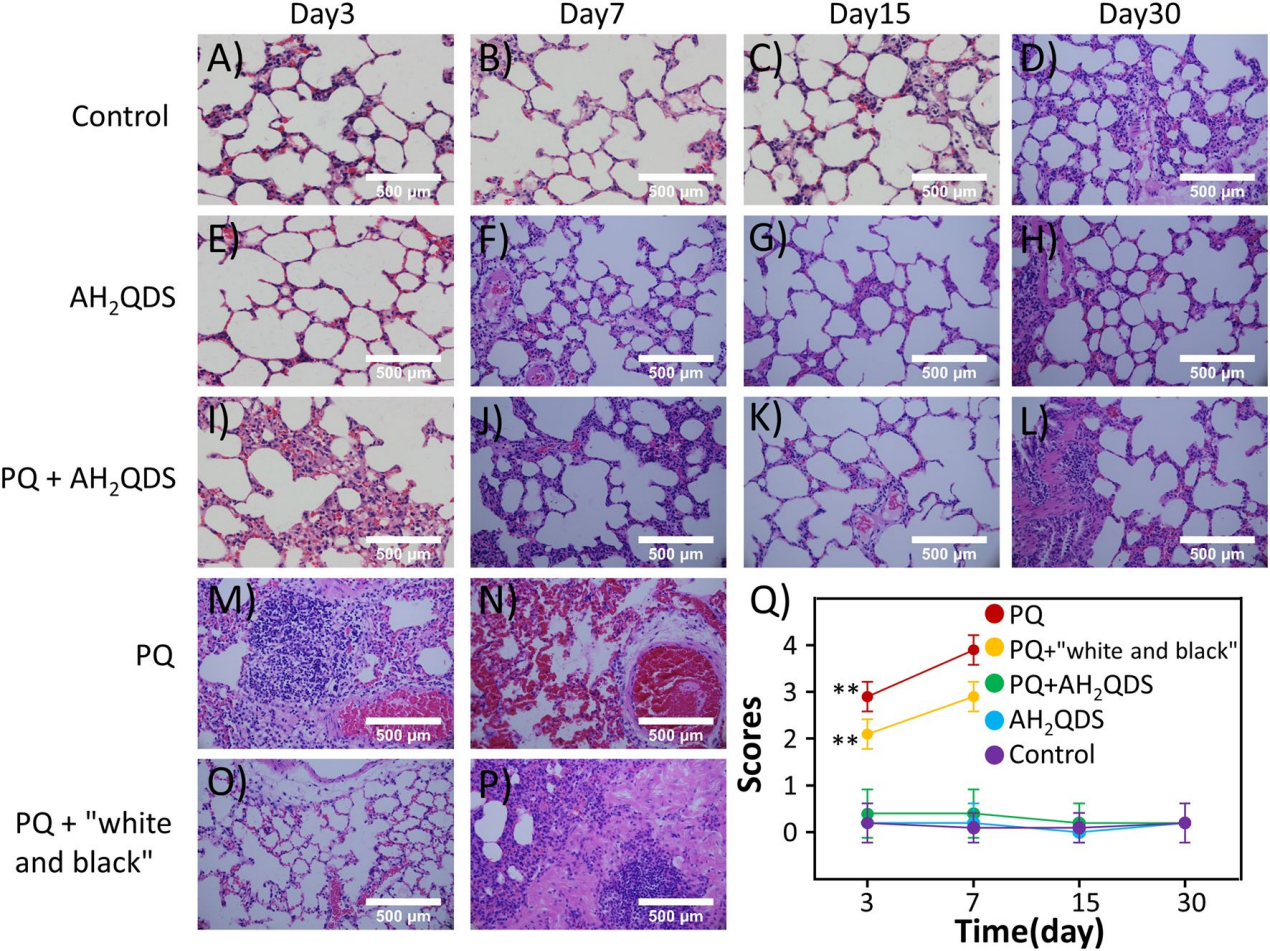
AH_2_QDS decreases lung injury. (**A**–**P**) H&E staining in lung tissue of rats in the diverse groups at different time points. (**A**–**D**) The untreated control group. (**E**–**H**) The AH_2_QDS group. (**I**–**L**) The PQ + AH_2_QDS group. (**M**,**N**) The PQ group. (**O**,**P**) The PQ + "white and black" group. Since a 4X LD50 dose of PQ was used, rats in the PQ group and PQ + "white and black" group died within one week, so there is no data for subsequent time points. (**Q**) Lung injury scores of different treatment groups. Data are presented as means ± SEM, n = 3, *P < 0.05, **P < 0.001.

The lung is the main target organ after PQ poisoning^30^. Patients often die of acute lung injury in the early stage, and pulmonary fibrosis often occurs later^31,32^. According to Fig. 5A–P, we also observed alveolar inflammation in the lung tissue of rats in the PQ and PQ + c "white and black" groups at all time points, including destruction of the alveolar structure, oedema in the alveolar cavity, intracapillary hyperaemia, and inflammatory cell infiltration, indicating acute lung injury. On the seventh day, alveolar fusion, alveolar septum thickening, and fibrous tissue hyperplasia were found in the lungs of the two groups, indicating pulmonary fibrosis. However, the lung tissues of rats in the untreated control, AH_2_QDS, and PQ + AH_2_QDS groups were as expected at all periods, with very little infiltration of inflammatory cells, no collapse of the alveolar walls, no thickening of the alveolar septa, no exudation in the alveoli, and no capillary dilation, hyperaemia or other manifestations. The pathological injury score of the lung tissue showed that lung injury in the PQ group and PQ + "white and black" group was significantly worse than that in the untreated control, AH_2_QDS, and PQ + AH_2_QDS groups, and the difference was statistically significant (p < 0.001) (Fig. 5Q).

**Figure 6:**
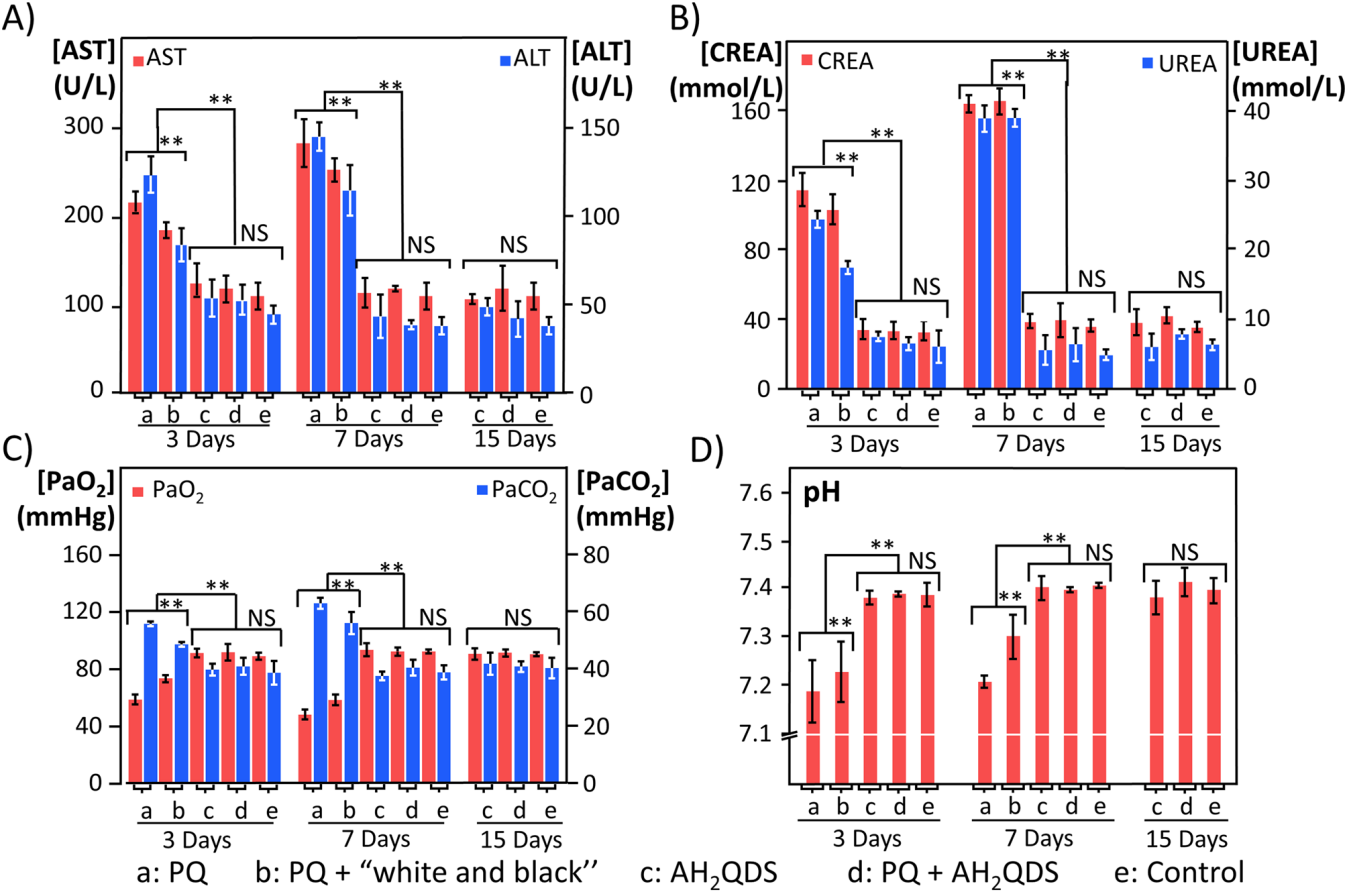
AH_2_QDS can minimize the damage of multiple organs and individual functions. (**A**,**B**) Changes in liver and kidney function of rats in each group. (**C**,**D**) Changes in blood gas analysis results of rats in each group. Due to a 4X LD50 dose of PQ intervention, animals in the PQ group and PQ + "white and black" group died within one week, so there is no subsequent time point data. At 30 days, all groups' liver and kidney function and blood gas results were in the normal range. Data are presented as means ± SEM, n = 3, NS = not significant, *P < 0.05, **P < 0.001.

In addition to lung injury, PQ poisoning can also cause severe functional damage to multiple organs, so we measured the liver and kidney function and blood gas of the SD rats in each group^33–35^. As shown in Fig. 6A,B, the ALT, AST, CREA, and UREA levels in the PQ and PQ + "white and black" groups increased from the 3rd day and peaked on the 7th day. The ALT, AST, CREA, and UREA levels in the untreated control, AH_2_QDS, and PQ + AH_2_QDS groups were significantly lower than those in the PQ and PQ + "white and black" groups in the first seven days, and the difference was statistically significant (P < 0.001). In addition, we compared the PQ group with the PQ + "white and black" group and found that the ALT, AST, CREA, and UREA levels in the PQ + "white and black" group were significantly lower than those in the PQ group (P < 0.001). However, the hepatic and renal function of the untreated control, AH_2_QDS, and PQ + AH_2_QDS groups was in the normal range during each period, and there was no significant difference between them (P > 0.05). PQ poisoning has been verified to cause functional damage to multiple organs, and AH_2_QDS treatment of PQ poisoning can alleviate liver and kidney function damage. The blood gas analysis results showed (Fig. 6 C-D) that the pH and PaO2 values in the PQ and PQ + "white and black" groups were significantly lower than those in the untreated control, AH_2_QDS, and PQ + AH_2_QDS groups (P < 0.001). Compared with the PQ group and PQ + "white and black" group, the pH and PaO2 values in the PQ + "white and black" group were significantly higher than those in the PQ group. The PaO2 values in the untreated control, AH_2_QDS, and PQ + AH_2_QDS groups were in the normal range during each period, and there was no significant difference between the three groups (P > 0.05). Contrary to this trend, the PaCO2 values in the PQ and PQ + "white and black" groups showed an increasing trend, indicating that hypoxaemia and carbon dioxide retention occurred in PQ-poisoned rats, which eventually led to type II respiratory failure. In summary, these findings suggest that AH_2_QDS can lower the damage to organ function caused by PQ poisoning.

### AH_2_QDS can rapidly decrease the concentration of PQ in vivo

**Figure 7:**
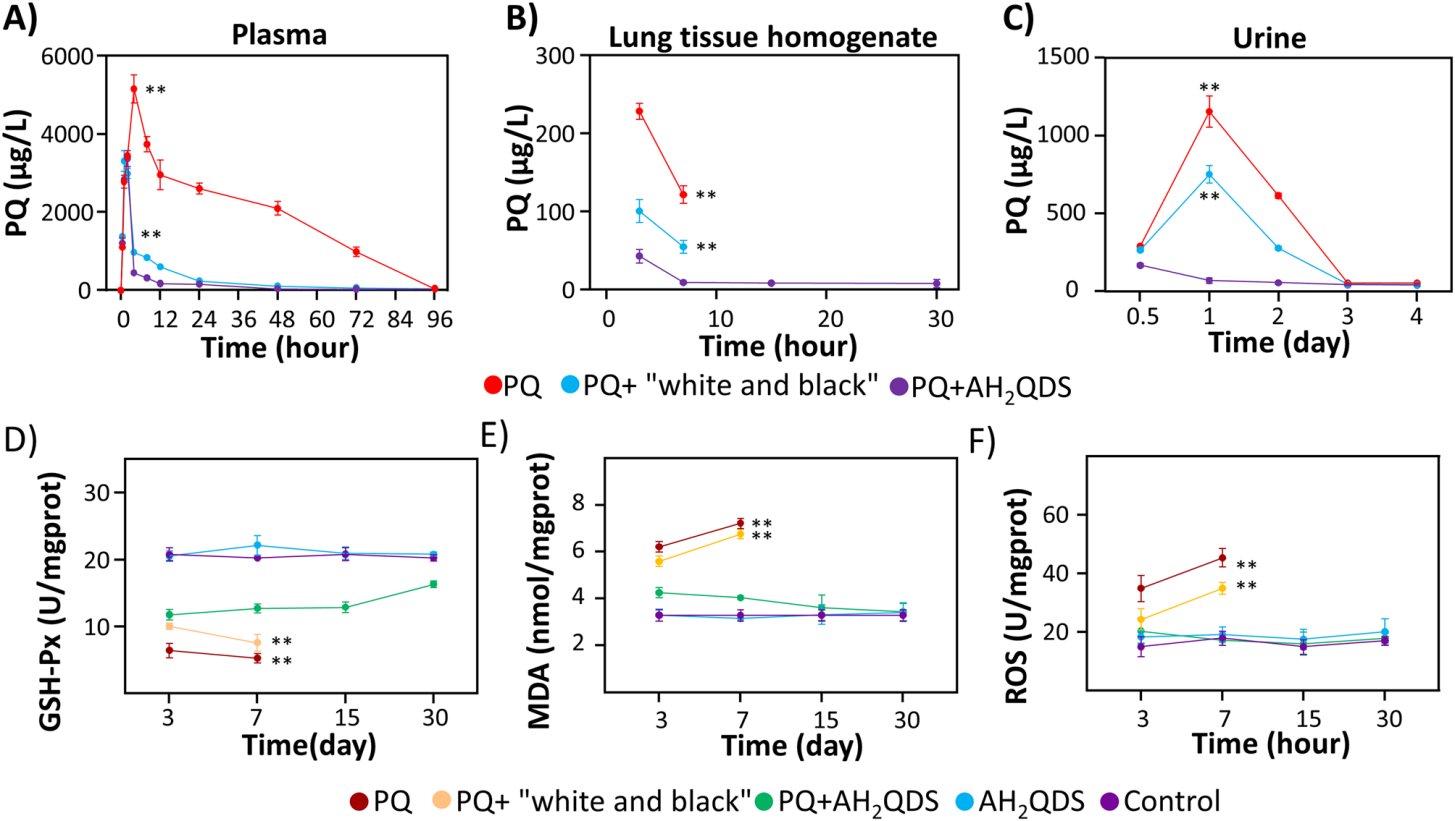
AH_2_QDS can decrease PQ drug concentration in vivo and improve the antioxidant reaction to oxidative stress. (**A**–**C**) The concentration of PQ in plasma, tissue and urine was detected by Ultra high-performance liquid chromatography-tandem mass spectrometry. (**D**–**F**) The levels of GSH-Px, MDA, and ROS were detected in different treatment groups. Due to a 4X LD50 dose of PQ intervention, animals in the PQ group and PQ + "white and black" group died within one week, so there is no subsequent time point data. Data are presented as means ± SEM, n = 3, *P < 0.05, **P < 0.001.

As shown in Fig. 7A–C, the PQ concentration in the PQ group peaked at 4 h and decreased to 0 at 96 h. The concentration of PQ in the PQ + AH_2_QDS group and PQ + "white and black" group decreased immediately after 2 h and was significantly lower than that in the PQ group (P < 0.001). The difference between 2 and 24 h was significantly smaller in the PQ + AH_2_QDS group than in the PQ + "white and black" group, and the difference was statistically significant. Similarly, the concentration of PQ in the lung tissue and urine decreased significantly in the PQ + AH_2_QDS group. The decrease in PQ drug concentration may have occurred because AH_2_QDS neutralizes PQ in the gastrointestinal tract.

### Protection of mitochondria by AH_2_QDS in vivo

**Figure 8:**
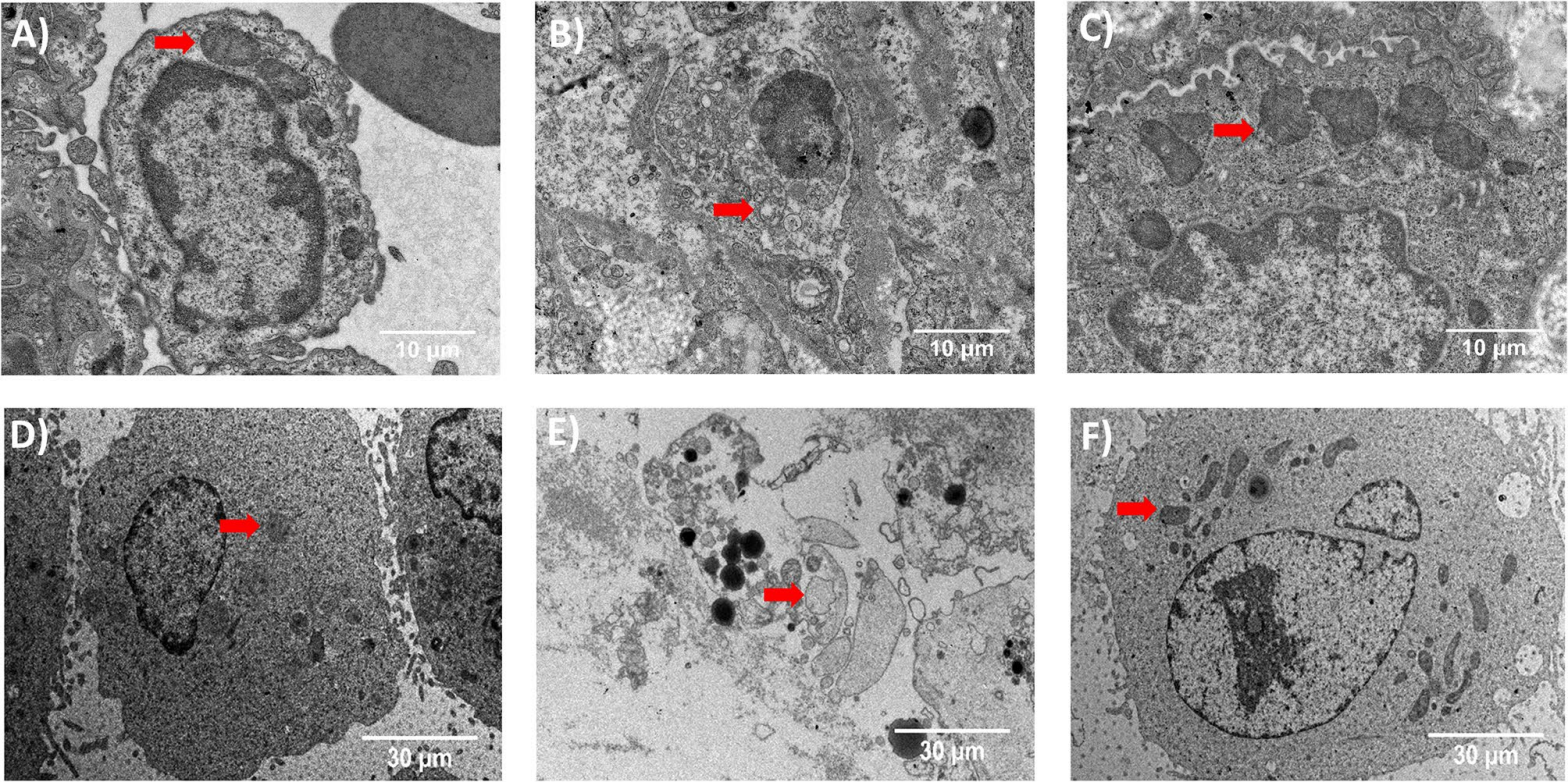
AH_2_QDS can protect mitochondria's structural integrity. (**A**–**C**) The transmission electron microscope observed mitochondria's structure in lung tissue. (**A**) the untreated control group did not receive any interventions. (**B**) PQ was given by gavage only at a concentration of 400 mg/kg in the PQ group. (**C**) in the PQ + AH_2_QDS group, 400 mg/kg of PQ was given to the stomach first, 2 h later, and 400 mg/kg of AH_2_QDS antidote was given. (**D**–**F**) The transmission electron microscope observed A549 cells' mitochondrial structure. (**D**) the untreated control group, without any treatment. (**E**) in the PQ group, A549 cells were incubated with 200 μm PQ. (**F**) A549 cells were incubated with 200 μM AH_2_QDS and 200 μM PQ in the PQ + AH_2_QDS group. The mitochondria have been marked with red arrows in the picture.

The induction of mitochondrial damage by PQ has been confirmed in in vitro experiments, and we also observed the same phenomenon in vivo experiments. Figure 8B showed that the PQ group's mitochondria were swollen, structurally damaged, vacuolated and empty. Under the electron microscope, the mitochondrial structure was as expected in the rat lung tissues in the untreated control group and PQ + AH_2_QDS group (Fig. 8A/C). These pictures illustrated that AH_2_QDS protects the structure of mitochondria.

### NextGen sequencing

**Figure 9:**
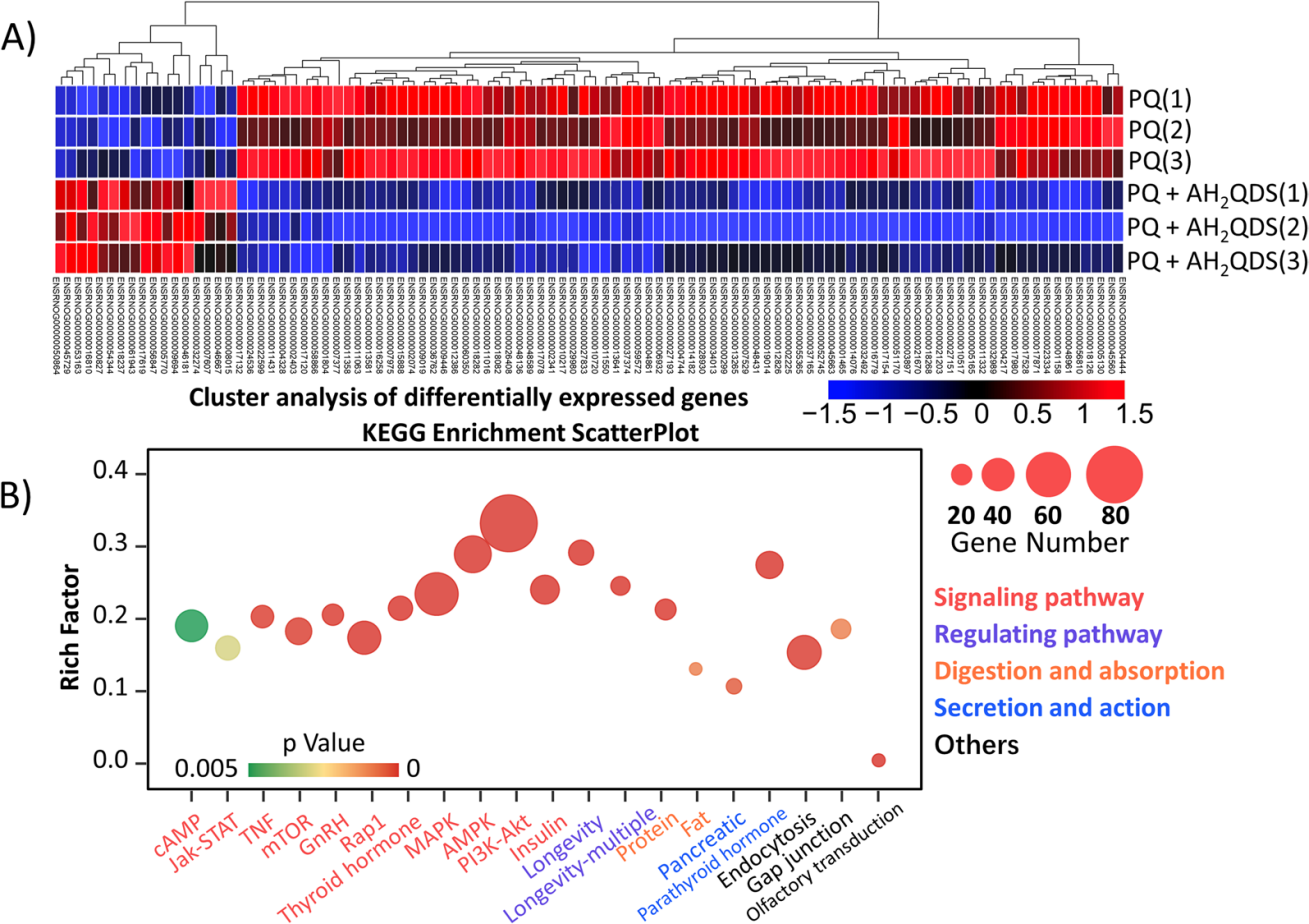
Differentially expressed genes by using NextGen sequencing method. (**A**) Heat map of differentially expressed gene expression profiles of the PQ group and the PQ + AH_2_QDS group. Gene enrichment analysis was performed based on differentially expressed genes, and the blue to red color indicates the expression level from low to high. (**B**) Regulatory biological pathways under AH_2_QDS treatment were analyzed using the KEGG database. Rich Factor indicates the proportion of differential genes annotated to this KEGG Pathway as a percentage of genome-wide genes. The horizontal coordinate indicates the description bar of the KEGG Pathway. GeneNumber, the number of genes annotated to this KEGG Pathway among the differential genes used for enrichment. The size of the dot indicates the number of genes on the enrichment, and the colour indicates the Qvalue, the lower, the more significant.

To better understand the detoxification mechanism of AH_2_QDS, we used RNAseq to investigate the differential gene expression patterns of rat lung tissue in the PQ and PQ + AH_2_QDS groups. Firstly, we performed data quality control (Figure S4A), after which we used principal component analysis (PCA) to identify outlier samples and high similarity samples. As illustrated in the Figure S4B, in this experiment, different samples from the same experimental group are arranged compactly and aggregated into clusters, showing good repeatability. In contrast, different experimental groups are clearly separated from each other, showing reasonable specificity. We can see from Fig. 9A that there were 3325 gene changes in the PQ group compared with the PQ + AH_2_QDS group, including 1455 upregulated genes and 1870 downregulated genes. As shown in Fig. 9B, the most differentially regulated pathways in these two samples are the PI3K-AKT pathway, MAPK pathway, AMPK pathway, etc. Consistent with our previous findings, these pathways are mainly oxidative stress-related pathways. We investigated the most significant pathway, namely, the PI3K-AKT pathway, to identify the genes with significant changes, and the results showed that Nrf2, Foxo3, Rxra, Itga4, Creb3l2, Angpt1, Egfr, Tnc, Lamc1, and Met were significantly upregulated. Nrf2 is significantly upregulated in tissues, and its function is closely related to oxidative stress, so we speculate that Nrf2 may be an essential gene for AH_2_QDS treatment of PQ poisoning.

**Figure 10:**
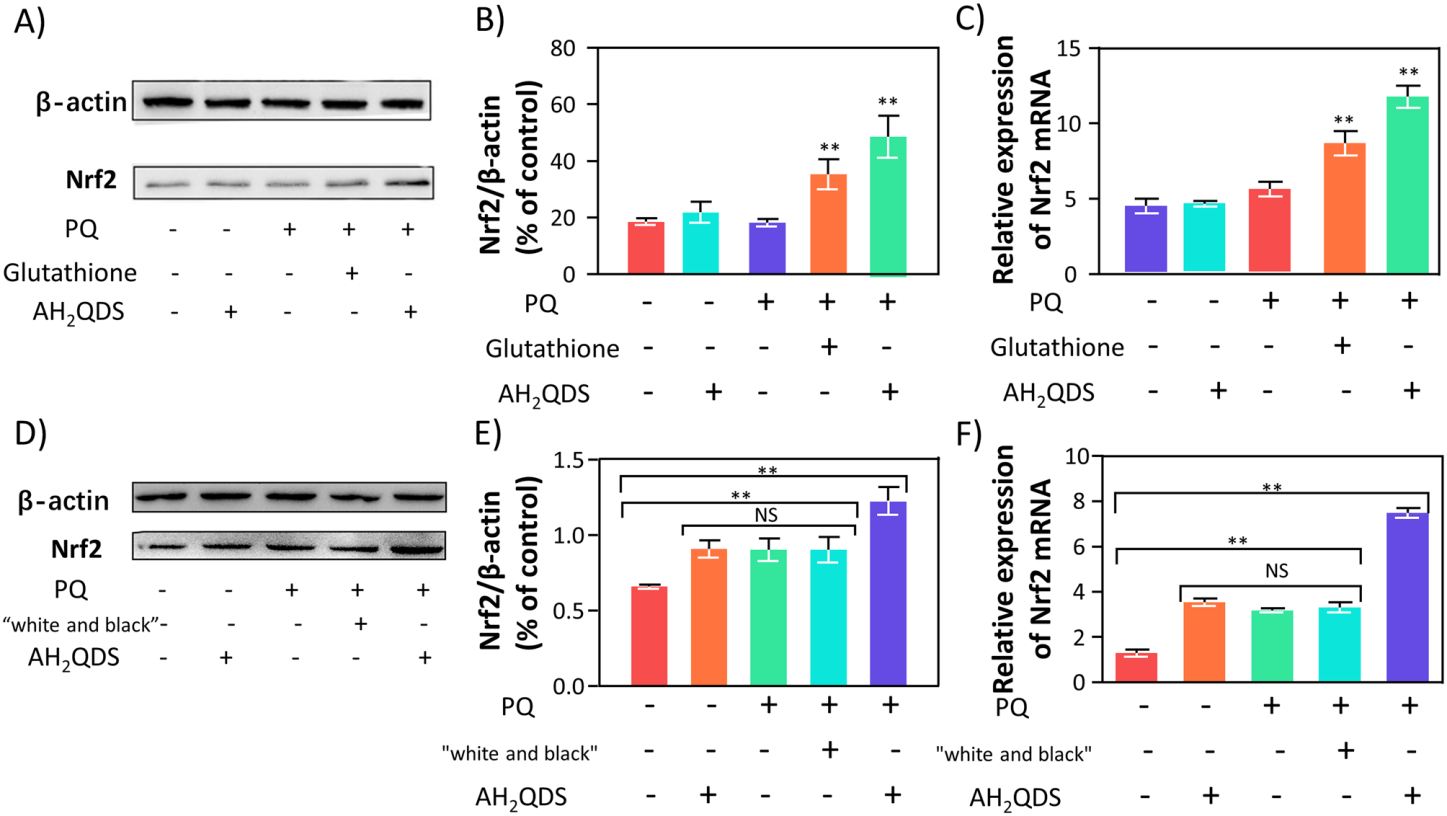
AH_2_QDS up regulated the expression of Nrf2. (**A**–**C**) After AH_2_QDS intervention, the expression of Nrf2 in A549 cells increased significantly. (**D**–**F**) The expression of Nrf2 in the lung tissue of rats detoxified by AH_2_QDS enhanced significantly. The grouping of gels/blots cropped from different parts of the same gel. Full-length gels and blots are included in Figure S6. Data are presented as means ± SEM, n = 3, NS = not significant, *P < 0.05, **P < 0.001.

We further verified by western blot and RT-qPCR experiments that in in vitro experiments, as illustrated in the Fig. 10A–C, compared with PQ treatment, glutathione and AH_2_QDS could significantly increase the expression of Nrf2, while the Nrf2 level of PQ + AH_2_QDS group was significantly higher than that of the PQ + glutathione group. The in vivo experiment showed that the levels of Nrf2 in the AH_2_QDS, PQ, and PQ + "white and black" groups were higher than that in the untreated control group, while the Nrf2 level in the PQ + AH_2_QDS group was significantly higher than those in the other groups (Fig. 10D–F). The results indicated that “white and black” scheme did not activate Nrf2. In contrast, glutathione could increase the expression of Nrf2, but its effect was weaker than that of AH_2_QDS, indicating that our antidote, AH_2_QDS, could significantly increase the expression of Nrf2, thus exerting its detoxification effect.

## Discussion

In this study, we used AH_2_QDS as an intervention in a rat model of PQ poisoning. Compared with those of the PQ group, the poisoning symptoms of the PQ + AH_2_QDS group were significantly improved, with a lower blood drug concentration, less organ function damage, and a higher survival rate. In the PQ + AH_2_QDS group, mitochondrial damage in lung tissue was alleviated, and a similar phenomenon was found in the cell test. The structure of the mitochondria was intact, the damage was significantly alleviated, and the expression of Nrf2 was significantly increased. These studies have proven for the first time that AH_2_QDS is an effective treatment for PQ poisoning, and Nrf2 plays a crucial role in its detoxification process.

Previous studies have shown that activated carbon or the “white and black” scheme can effectively treat PQ poisoning^6–8^. In contrast, our experiments only confirmed that the conventional “white and black” scheme can quickly and effectively degrade the PQ blood concentration but does not affect the survival rate of rats. Specifically, a 4X LD50 dose of PQ was used to construct the poisoning model, and the drug intervention time was as long as 2 h, during which most of the PQ may have been absorbed into the blood, while the “white and black” scheme could only absorb the residual poison in the stomach and accelerate its excretion but had no effect on the PQ already in the blood. Additionally, the results also showed that AH_2_QDS is not only faster than the “white and black” scheme in removing toxins but also plays a specific role in the blood-related effects of PQ.

The toxic effect of PQ on mitochondria was proposed as early as 1968^36^. Since then, a large number of studies on the damage of PQ to mitochondria have been published^26–28^. Some scholars indicated that PQ could cause accumulation of the hMn-SOD precursor of human manganese-dependent peroxidase and diminish Mn-SOD activity. The conversion of GSH to GSSG leads to a decrease in GSH levels and weakens its antioxidant activity^37^. Other studies have shown that PQ can cause the production of H_2_O_2_ and lessen the activity of catalase^38^. H_2_O_2_ can induce changes in mitochondrial permeability and affect the mitochondrial membrane potential, resulting in the movement of cytochrome C from the mitochondria into the cytoplasm, and then induce apoptosis by activating caspase9^39^. In our study, the microstructure of the lung tissue and A549 cells in the PQ group was observed under a projection electron microscope. It was found that the structure of the mitochondria was destroyed, vacuoles appeared in the cells, the cell walls were broken, and a large number of organelles were extruded. In contrast, the morphology of the mitochondria in the PQ + AH_2_QDS group was as expected, and the lamellar structure was normal. The cell membrane potential of the PQ + AH_2_QDS high-dose group was the highest, and the membrane potential was positively correlated with the concentration of AH_2_QDS, while the mitochondrial membrane potential of cells treated with only PQ was the lowest. In summary, PQ can destroy the tissue structure of the mitochondria, affect the membrane potential, and eventually lead to cell rupture and death, while AH_2_QDS can prevent this process and protect the function and structure of the mitochondria.

The data show that after PQ is absorbed into the blood, it causes the formation of excess reactive oxygen species (ROS), which leads to imbalance of the redox system, the consumption of NADPH, damage to mitochondria, the destruction of lipids, proteins and DNA, and a decrease in the activity of various antioxidant enzymes^24^. After continuous oxidative stimulation, the body eventually sustains tissue damage. A large amount of ROS produced by PQ may be the leading cause of acute lung injury caused by PQ poisoning. In this study, it was found that after PQ exposure, the levels of ROS and MDA in the PQ group and conventional treatment group increased, while the level of GSH-Px decreased. In the PQ + AH_2_QDS group, the ROS and MDA levels decreased, and the level of GSH-Px increased. The results show that PQ can produce a large amount of ROS to cause lipid peroxidation and oxidative stress injury. AH_2_QDS can inhibit PQ's effect, improve antioxidant ability, and decrease the level of lipid peroxidation.

After further investigation of the detoxification mechanism of AH_2_QDS, we found that the main differentially regulated pathway was the oxidative stress pathway, in which we found that the nuclear factor Nrf2 was significantly upregulated. Many studies have shown that Nrf2 can be used as a "guard" to protect the body against a variety of toxic effects^40–42^. Nrf2 can be activated in a variety of processes involving oxidative stress. Nrf2 was expressed in epithelial cells, macrophages and vascular endothelial cells of normal rat lung tissue^43–45^. MDA in the serum of rats poisoned by PQ increased significantly with the prolongation of poisoning time, while the activity of SOD decreased significantly. Nrf2 protein increased significantly in lung tissue injury induced by PQ. It has been found that the Nrf2-ARE pathway protects the lungs against dibutyl hydroxytoluene-induced acute respiratory distress syndrome (ARDS) and hyperoxia-induced lung injury by activating antioxidant enzymes^46,47^. In our experiment, PQ, as a potent stressor, could activate the Nrf2 signalling pathway. Nrf2 was expressed at low levels in normal rat lung tissue and A549 cells, but the expression of Nrf2 was significantly increased after AH_2_QDS treatment. These results show that Nrf2 plays a vital role in the treatment of PQ poisoning by AH_2_QDS.

The direct mechanism of AH_2_QDS in the treatment of PQ poisoning is that AH_2_QDS enters the gastrointestinal tract and comes into contact with the PQ solution. Through a rapid redox reaction, PQ is reduced to a nontoxic green needle-like solid. Thus, detoxification is realized. Energy spectrum analysis showed that the acicular substance was stable and could not be dissolved in strong acids, strong bases, or organic solvents and was extremely stable at room temperature and pressure. At the same time, it was also found in the faeces of SD rats. We were concerned that after administration of AH_2_QDS, a green needle-like solid will be formed in the blood, leading to the formation insoluble thrombi and resulting in thrombotic disease and a series of clinical symptoms. Therefore, we tested the blood and tissues of experimental animal SD rats but did not find this substance. So, after administering AH_2_QDS, green needle-like solids would not be formed in the blood, tissues and organs to cause thrombotic disease.

To prove whether there is an indirect mechanism of AH_2_QDS in PQ poisoning treatment, we constructed animal and cell models. ELISA, WB, and qPCR were performed to detect the levels of GSH-Px, MDA, ROS, and Nrf2, and transmission electron microscopy was performed to observe the microstructure of the mitochondria. The same trend was observed in vivo and in vitro. After the intervention with AH_2_QDS, the expression of nuclear factor Nrf2 was enhanced, mitochondrial damage was relieved, and antioxidant reaction to oxidative stress was improved. Unfortunately, our experiment cannot determine whether the mechanism of AH_2_QDS in the treatment of PQ poisoning is the direct mechanism or the indirect mechanism. Further research is needed.

In this paper, AH_2_QDS was used as an antidote in the treatment of PQ poisoning for the first time and achieved excellent results, but this was verified only in SD rats, and it has not been tested in more advanced mammals; thus, a long and strict clinical study is needed to investigate the use of AH_2_QDS in humans. Additionally, a 4X LD50 dose of PQ was given to SD rats in the poisoning model, and AH_2_QDS was given for detoxification 2 h later. The 30-day survival rate of SD rats in the treatment group reached 100%, but if the time window of treatment with AH_2_QDS were prolonged (2.5 h, 3 h, 4 h, or 6 h), the 30-day survival rate of SD rats in the treatment group decreases with the prolongation of intervention time. This may be because 2 h after ingestion of PQ, the rats have rapidly absorbed it into the blood and transported it to various organs through the blood flow. Even if AH_2_QDS can detoxify the absorbed PQ, too high a concentration of PQ causes irreversible toxic damage to the organs in this time. In the follow-up studies, the sequencing results will be further analysed, and mechanistic research will be performed to elucidate the molecular functions of the gene, the cell location, and the biological process involved. At the same time, experiments were carried out on the Nrf2-ARE pathway through gene silencing/overexpression of related proteins to demonstrate the profound relationship between the Nrf2-ARE pathway and AH_2_QDS in the treatment of PQ poisoning.

## Conclusion

In summary, paraquat poisoning is still an extremely high clinical mortality disease, and conventional treatments are clinically ineffective. The new antidote we developed, AH_2_QDS, can lower the concentration of PQ by binding it and protect the mitochondria and reduce the oxidative stress damage caused by PQ. The relationship between mitochondrial damage, the expression changes upstream and downstream of the Nrf2-ARE pathway, and AH_2_QDS in PQ poisoning treatment must be further explored.

## Methods

### Animals and cell lines

All animal experiments were performed as per the protocols approved by the Animal Care and Use Committee of Hainan Medical University. All methods were performed in accordance with the guidelines and regulations of the Animal Care and Use Committee of Hainan Medical University and as per the ARRIVE guidelines 2.0. Human type II alveolar lung epithelial cells (A549) were purchased from the Shanghai Institute for Biological Sciences. The cells were maintained in a 5% CO2 incubator at 37 °C in medium (F12K) supplemented with 10% FBS and penicillin/streptomycin (100 U/ml). Sprague–Dawley (SD) rats (8 weeks, male, SPF grade) were purchased from Changsha Tianqn Biotechnology Co., Ltd., and were maintained in specific pathogen-free (SPF) facilities.

### Main reagent

Paraquat solution, purchased from Nanjing Red Sun Co., Ltd., was given at a 400 mg/kg concentration. Twenty percent PQ solution was diluted into 1 mL of PQ solution with PBS. For the “white and black” scheme, 500 mg/kg activated carbon, 500 mg/kg montmorillonite powder and 5 mL mannitol were used for gastric cancer. Activated carbon was purchased from National Pharmaceutical Group Chemical Reagent Co., Ltd. Montmorillonite powder was purchased from Xiansheng Pharmaceutical Co., Ltd. Mannitol (20%) was purchased from Jiangsu Zhengda Tianqing Pharmaceutical Co., Ltd. Anthrahydroquinone-2-6-disulfonate (AH_2_QDS) was synthesized by the Chinese Academy of Tropical Agricultural Sciences. The method is patented (Patent No: 2016103413306). Chemical name: anthraquinone-2-dioxo-6-disodium disulfonate, chemical formula: C14H8O8S2.2Na, molecular weight: 368.33. We prepared the AH_2_QDS solution at a concentration of 40 mmol/L.

### Modeling studies of PQ and AH_2_QDS binding

Chemical structures of PQ and AH_2_QDS were drawn with ChemDraw Pro 16.0 software. The binding conformations between PQ and AH_2_QDS were simulated with AutoDock Vina^20^.

### Cell counting kit-8 (CCK8)

A549 cells were incubated with different concentrations of PQ, AH_2_QDS and glutathione for 12 h. After 12, 24, 48 and 72 h, 10 μL of CCK8 solution (Dojindo, Japan) was added, and the cells were incubated in the incubator for 2 h. An enzyme labelling instrument was used to measure the absorbance at 450 nm, and a formula was used to calculate the cell viability.

### Mitochondrial membrane potential

The cell culture medium was removed, the cells were washed with PBS, 1 ml of medium was added, and 1 mL of JC-1 staining working solution was added and mixed well. After incubating the cells for 20 min in the incubator at 37 °C, the supernatant was removed, the cells were washed with diluted staining buffer (1x), 2 mL of medium was added, and images were captured under the fluorescence microscope.

### Animal experiments

SD rats (~ 300 g) were subjected to gavage 400 mg/kg PQ, and 500 mg/kg "white and black" scheme and 400 mg/kg AH_2_QDS intervention treatment were administered 2 h later. The specific methods used to establish the model is shown in Figure S3. We selected rats without collecting blood after establishing the model and observed and recorded the survival of each group over 30 days. The occurrence of death was recorded as 1, and no death was recorded as 0. Finally, a survival curve was drawn. The animal protocol passed the ethical review of the ethics committee of The First Affiliated Hospital of Hainan Medical University. (Issue number: 2020 (Research) No. (97); Review category: A Quick Review; Decision: Approval; Decision Date: July 8, 2020).

### Sample collection

Blood was collected from the rats at different time points in anticoagulant tubes treated with heparin, and the plasma was separated and stored at − 80 °C for the detection of drug concentrations in the blood. The urine was left in the centrifuge tube for the detection of drug concentrations in urine. Rats were anaesthetized by intraperitoneal injection of 10% chloral hydrate (300 mg/kg), and the blood from the abdominal aorta was collected for the detection of liver, kidney and lung function. Finally, the rats were killed by exsanguination, and the lung tissue was collected, washed with PBS and stored at − 80 °C for follow-up analysis. The bodies of the animals were then incinerated.

### Histopathology

SD rats were sacrificed at different times, and the lungs of the rats were harvested, fixed in 4% formalin, embedded in paraffin, sectioned, and stained with haematoxylin and eosin (H&E). The lung injury score was determined according to methods that were previously reported in the literature^48^. A score of 0 means there is no alveolitis. 1 point means mild alveolitis, the lesions are limited to local and pleural lesions, accounting for less than 20% of the lung, and the alveolar structure is sound. A score of 2 indicates moderate alveolitis, and the lesion area accounts for 20–50% of the lung. Finally, a score of 3 means severe alveolitis, with diffuse alveolitis involving more than 50% of the lung.

### Blood analysis

The collected venous blood samples were placed into a test tube with a coagulant and centrifuged at 3000 r/min for 5 min. Rat serum was obtained and placed into an automatic biochemical function analyser for analysis. After collecting blood from the abdominal aorta with an arterial blood gas sampler and rubbing with both hands for 1 min, 0.1 mL was injected into the blood gas analyser for analysis.

### Transmission electron microscopy

Lung tissue and A549 cells were collected and placed overnight in 2.5% glutaraldehyde fixed solution that was prechilled at 4 °C, cleaned with PBS, fixed with 1 ml of 1% osmic acid for 1.5 h, dehydrated with alcohol and acetone, and impregnated with resin, and ultrathin sections were stained with uranium acetate and lead citrate. The ultrastructure was observed under a transmission electron microscope.

### Ultra-high-performance liquid chromatography-tandem mass spectrometry

The concentrations of PQ and AH_2_QDS were determined by ultra-high-performance liquid chromatography-tandem mass spectrometry (UPLC/Xevo TQ-S, Waters). The mobile phase was acetonitrile/100 mM ammonium formate (pH = 3.7) = 50 × 50, and the flow rate was 0.3 mL/min. An ACQUITY UPLC BEH HILIC column (100 mm × 2.1 mm, 1.7 μm) was used. PQ was quantified in the MRM mode of positive ion multireaction monitoring with an electrospray ion source. Negative ion SIR mode was used to quantify AH_2_QDS. The parameters were as follows: capillary voltage: 3.2 kV, ion source temperature: 150 °C, cone hole back blowing gas flow rate: 30 L/hr, dissolvent temperature: 350 °C, and dissolvent gas flow rate: 800 L/hr.

### Cytokine detection

The GSH-Px, MDA and ROS kits purchased from Nanjing Jiancheng Company were used according to the instructions to detect the levels of GSH-Px, MDA and ROS, respectively.

### Western blotting

Proteins were extracted from tissues and cells with a BCA kit (Biyuntian Biotechnology Co., Ltd.), separated in SDS-PAGE gels, and transferred to cellulose membranes. After sealing, the membranes were incubated with the primary antibody overnight, then incubated with the secondary antibody for 1 h (Table S1), and finally developed by exposure.

### Quantitative real-time polymerase chain reaction (RT-qPCR)

TRIzol (Biyuntian Biotechnology Co., Ltd.) was used to extract RNA, and a cDNA reverse transcription kit (Applied Biosystems, cat. no. 4368814) was used to reverse-transcribe the extracted RNA into cDNA. PCR was performed on an ABI Prism 7900HT system (Applied Biosystems, Foster City, CA, USA) using SYBR GREEN PCR Master Mix (Applied Biosystems). Primers were purchased from Sangon Biotech (Shanghai) Co., Ltd. The primer sequences are listed in Table S2.

### NextGen sequencing

Total RNA was extracted from rat lung tissue in the PQ and PQ + AH_2_QDS groups and enriched with eukaryotic mRNA using magnetic beads with Oligo(dT). The second cDNA strand was then purified by QiaQuick PCR kit and eluted with EB buffer, followed by end repair, the addition of poly(A) and ligation of the sequencing junction, then agarose gel electrophoresis for fragment size selection, and finally PCR amplification. After that, the library was sequenced on the Illumina NovaSeq6000 platform.

To make sure reads reliable, Illumina paired-ended sequenced Raw reads were filtered using the fastp to remove low quality reads (https://github.com/OpenGene/fastp). The filtered data is then compared to the reference sequence. Reference genome and gene model annotation files were downloaded from genome website directly. (https://www.ncbi.nlm.nih.gov/assembly/GCF_000001895.5#/def). The sequenced data were imported into Partek Flow (Partek Inc., St. Louis, MO) and principal component analysis (PCA) images were generated to visualise distribution differences.

Differential expression analysis was performed using the DESeq2^49^. Based on the Kyoto Encyclopedia of Genes and Genomes (KEGG)^50^, we used the R package cluster Profiler^51^ to perform KEGG functional enrichment analysis of differentially expressed genes.

### Statistical analysis

Statistical analyses were performed using GraphPad Prism 8.0 or SPSS 20.0 software. Measurement data are expressed as the mean ± SEM, and significance was tested by single-factor analysis of variance (ANOVA). Kaplan–Meier survival analysis was used to analyse the survival rate of rats in different treatment groups. P < 0.05 indicates that a difference is statistically significant.

### Ethical approval

The experiment was carried out according to the guiding principles for animal experiments at Hainan Medical University.

## Supplementary Information

Supplementary Information.
