# Supplementary material for: Anthrahydroquinone-2-6-disulfonate is a novel, powerful antidote for paraquat poisoning
Source: Sci Rep. 2021 Oct 11;11:20159. doi: 10.1038/s41598-021-99591-4 (PMC8505516; doi:10.1038/s41598-021-99591-4)
Supplement: Supplementary file 1 — Supplementary Information. [file 41598_2021_99591_MOESM1_ESM.docx]

**Supplementary Material**

**Newly discovered a powerful antidote for paraquat poisoning**

**Jin Qian^1#^, Chun-Yuan Wu^2#^, Dong-Ming Wu^2^, Li-Hua Li^1^, Qi Li^1^, Tang Deng^1^, Qi-Feng Huang^1^, Shuang-Qin Xu^1^, Hang-Fei Wang^1^, Xin-Xin Wu^1^, Zi-Yi Cheng^1*^, Chuan-Zhu Lv^3*^****, Xiao-Ran Liu^1*^**

*1* *Key Laboratory of Emergency and Trauma of Ministry of Education, The First Affiliated Hospital of Hainan Medical University, Hainan Medical University, 571199, Haikou, China*

*2Institute of Environment and Plant Protection, Chinese Academy of Tropical Agricultural Sciences, 571101, Haikou, China*

*3* *Emergency Medicine Center, Sichuan Provincial People's Hospital, University of Electronic Science and Technology of China, 610072, Chengdu, China*

#These authors contributed equally to this work.

*Corresponding authors: Zi-Yi Cheng, chengziyi@hainmc.edu.cn; Chuan-Zhu Lv, lvchuanzhu677@126.com; Xiao-Ran Liu, hy0203049@hainmc.edu.cn;

**Figure S1.** In vitro cytotoxicity-test. A) Different concentrations of paraquat interfered with the changes of cell viability of A549 cells. B) Changes of cell viability of A549 cells treated with different concentrations of AH2QDS at 72 h. C) At 72 h, the cell viability of A549 cells treated with different concentrations of glutathione changed.


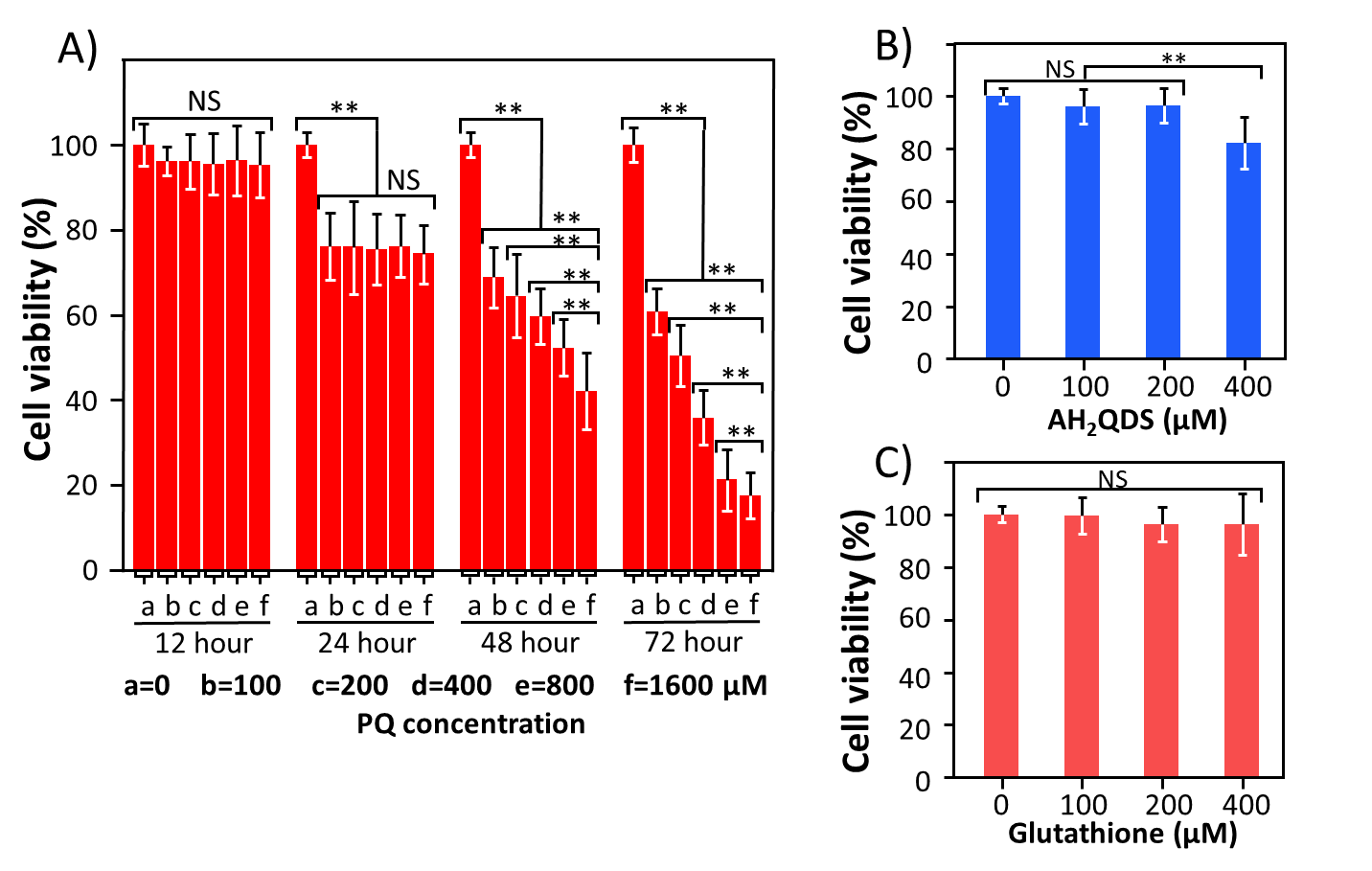


**Figure S2.** Detoxification of pretreated AH2QDS to PQ poisoning in vitro. A549 cells were pretreated with 200 μM AH2QDS / Glutathione for 1h, 6h, 12h and 24h. The cells were given 200 μM PQ before being incubated for 72h to observe changes in cell viability. Data are presented as means ± SEM, n = 3, NS = not significant, *P<0.05, **P<0.001.


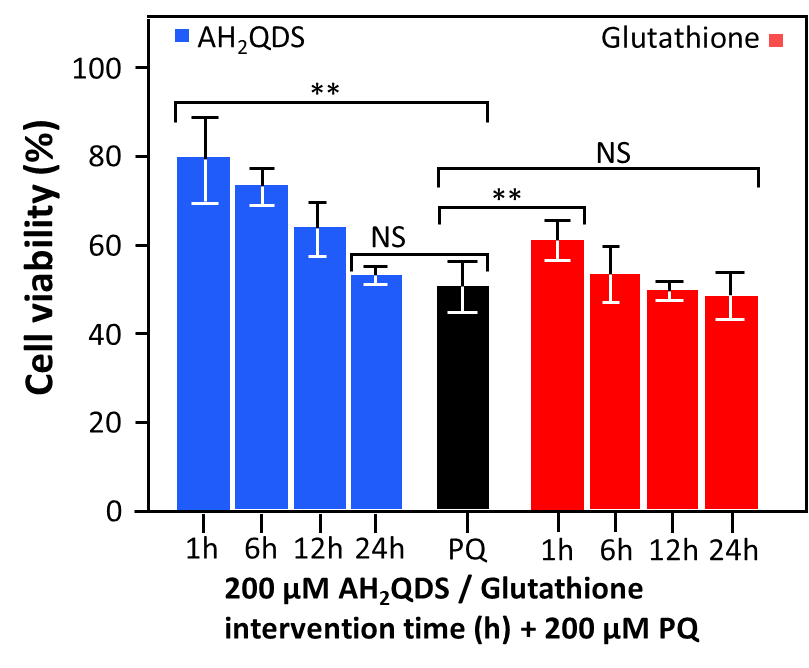


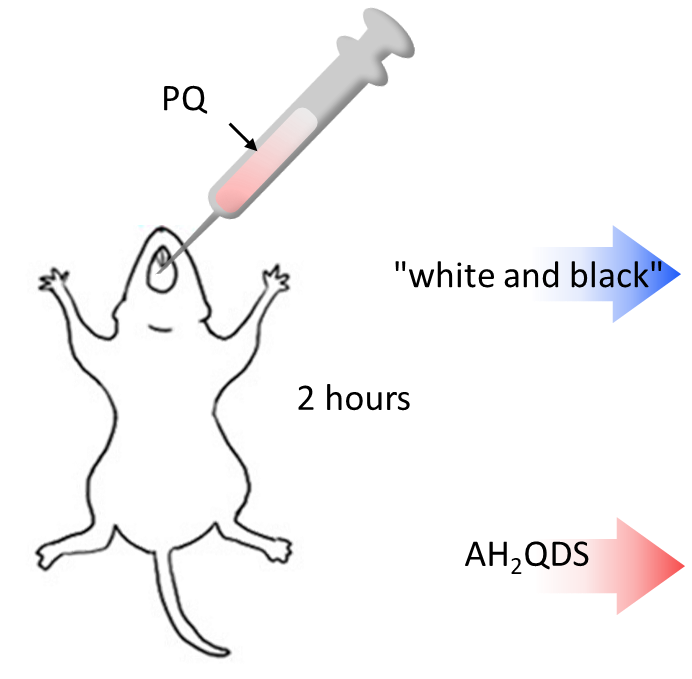
**Figure S3.** Scheme describing the administration ways.


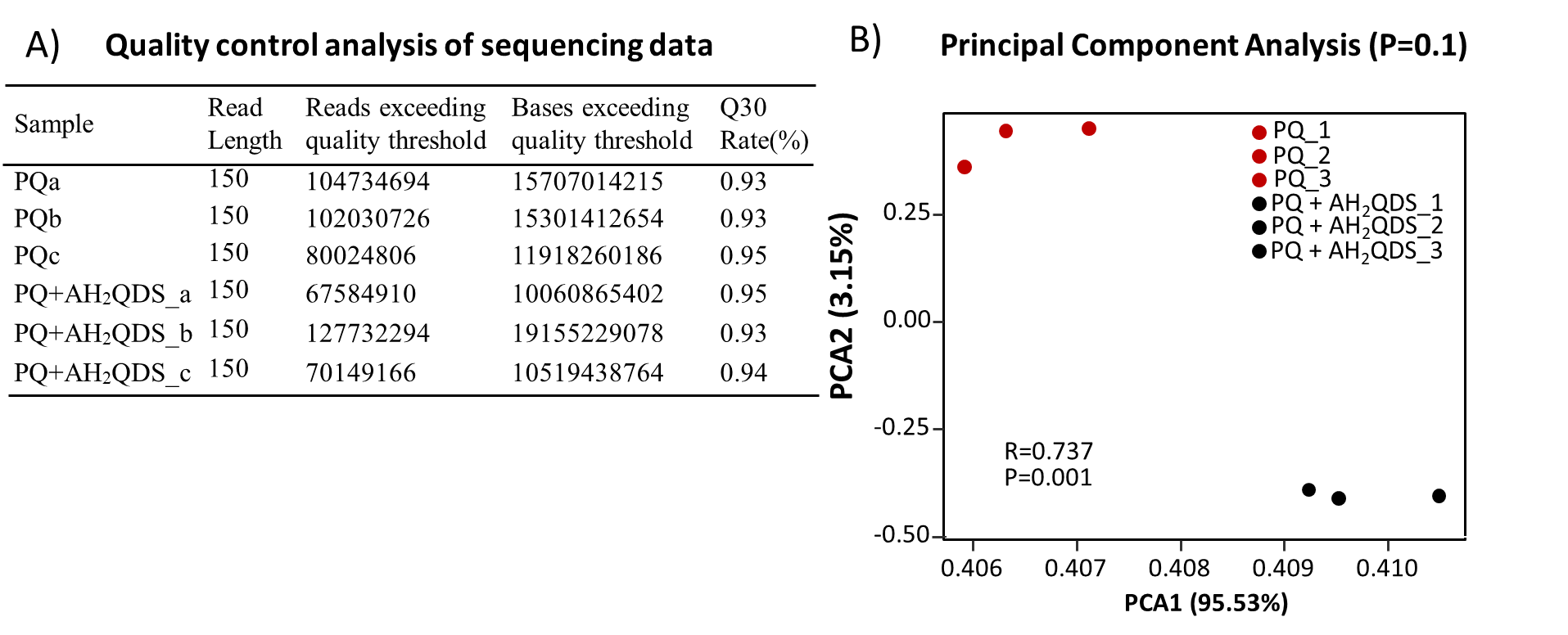
**Figure S4.** A) Quality control analysis of sequencing data. Q30 indicates a sequencing error rate of 0.1%. B) Principal Component Analysis. We determined the first principal component (PC1) and second principal component (PC2) of gene expression differences between samples using the gene expression of each sample as the basis and plotted the position of each sample (points of different shapes) on the axes, and the clustering relationship of the samples could be seen from the distance between samples in terms of distance.


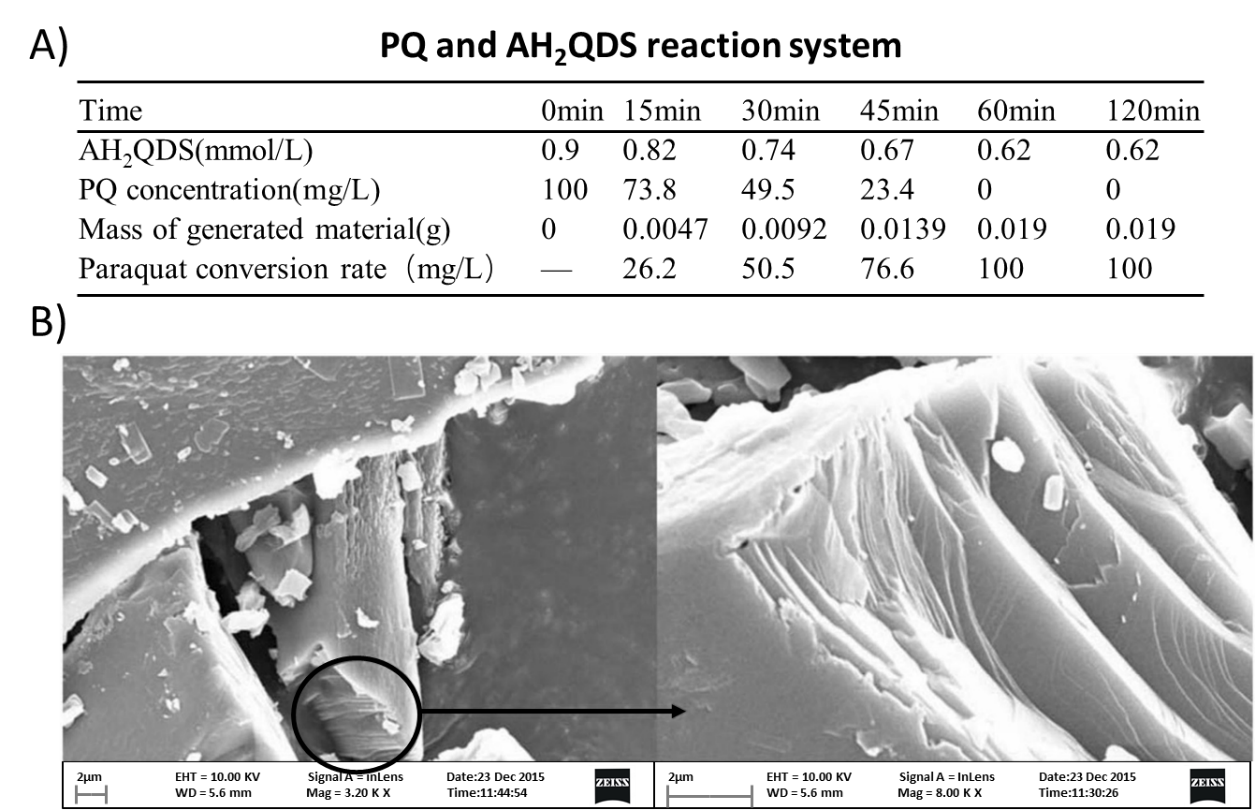
**Figure S5**. A) PQ and AH_2_QDS reaction system. Configure paraquat solution with a concentration of 200mg/L PQ (pH=6), take 50mL into a Xilin bottle, inflate with high purity nitrogen for 5min, make the dissolved oxygen concentration 1%, cover with rubber cap immediately after inflation and seal with pressurized aluminium cap, use syringe, draw AH_2_QDS 50mL was injected into paraquat solution by puncturing the aluminium cap of the syringe, and the mass of PQ, AH_2_QDS and reaction products were measured at different time points. B) Scanning electron microscope (SEM) observation of the structure of the reaction products.

**Table S1.** Antibody information of Western blotting

| Types | dilution | brand | number |
| --- | --- | --- | --- |
| Nrf2 (primary) | 1:500 | Abcam | ab89443 |
| β-actin (primary) | 1:25000 | Abcam | ab49900 |
| Goat Anti-Mouse IgG H&L (secondary) | 1:2000 | Abcam | ab205719 |

**Table S2.** The primer sequences.

|  | upstream primer (5'-3') | downstream primer (5'-3') |
| --- | --- | --- |
| Nrf2 | GCCTTCCTCTGCTGCCATTAGTC | TCATTGAACTCCACCGTGCCTTC |
| β-actin | CCAAGGCCAACCGCGAGAAGATGAC | CCAAGGCCAACCGCGAGAAGATGAC |

**Figure S6.** Full-length gels and blots


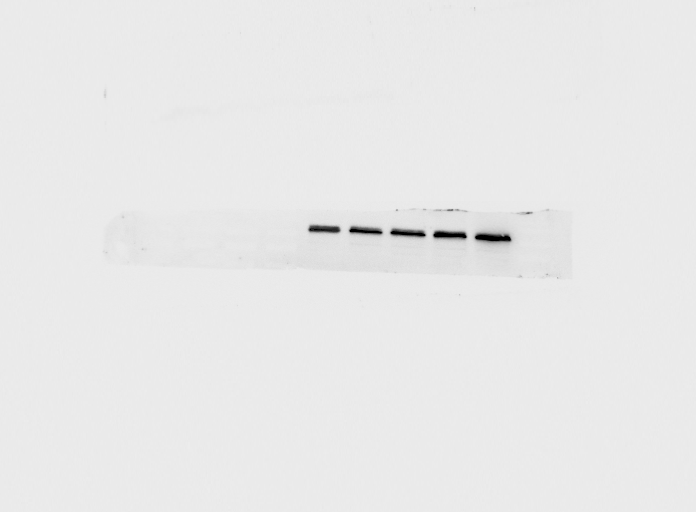


A549 cell，β-action，western bolt


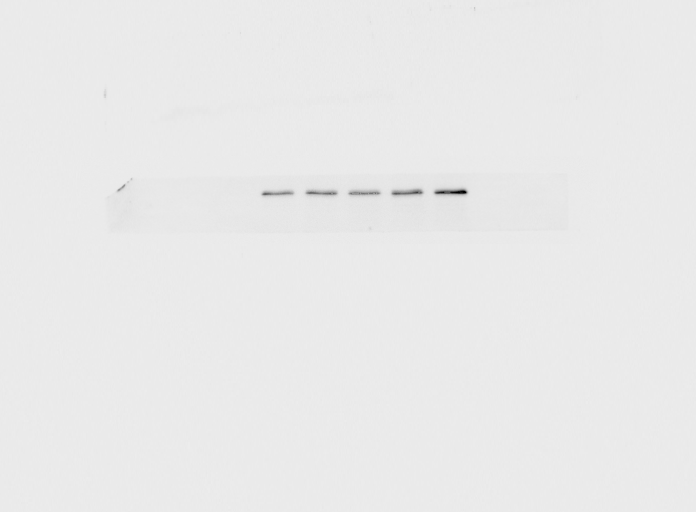


A549 cell，Nrf2，western bolt


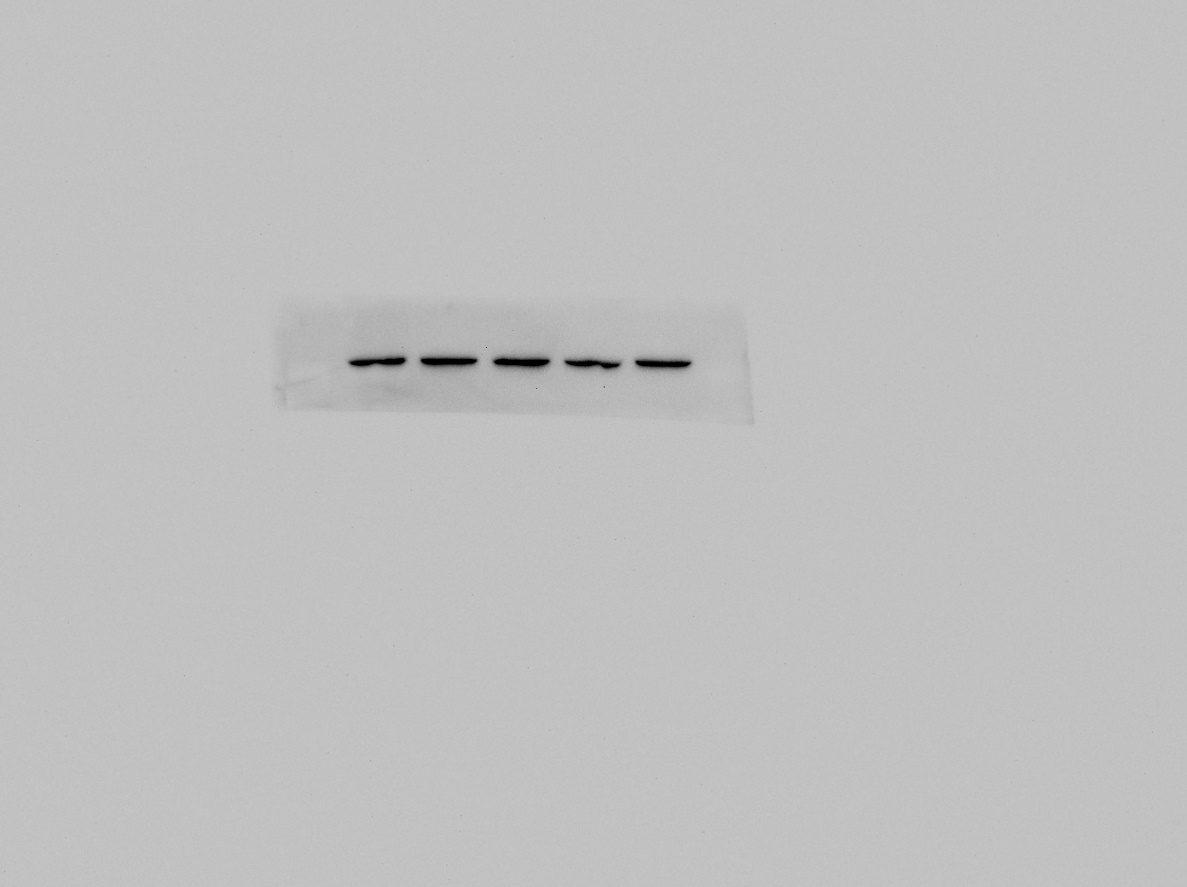


rat，β-action，western bolt


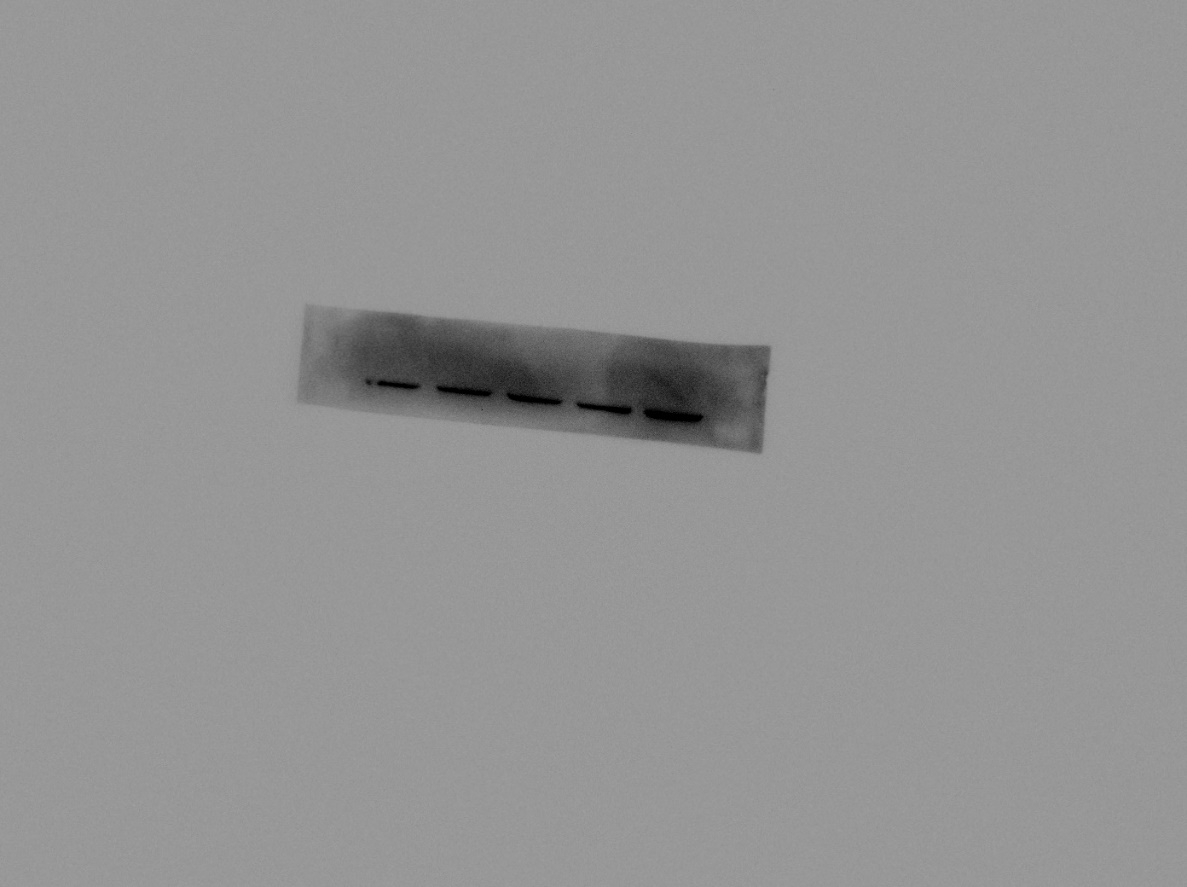


rat，Nrf2，western bolt
